# Impact of primary care provider density on detection and diagnosis of cutaneous melanoma

**DOI:** 10.1371/journal.pone.0200097

**Published:** 2018-07-13

**Authors:** Nathaniel H. Fleming, Madeline M. Grade, Eran Bendavid

**Affiliations:** 1 Department of Health Research and Policy, Stanford University School of Medicine, Stanford, California, United States of America; 2 Division of Primary Care and Population Health, Department of Medicine, Stanford University, Stanford, California, United States of America; 3 Center for Health Policy and the Center for Primary Care and Outcomes Research, Stanford University, Stanford, California, United States of America; Universidade de Sao Paulo, BRAZIL

## Abstract

**Introduction:**

Early diagnosis of cutaneous melanoma is critical in preventing melanoma-associated deaths, but the role of primary care providers (PCPs) in diagnosing melanoma is underexplored. We aimed to explore the association of PCP density with melanoma incidence and mortality.

**Methods:**

All cases of cutaneous melanoma diagnosed in the United States from 2008–2012 and reported in the Surveillance, Epidemiology, and End Results (SEER) database were analyzed in 2016. County-level primary care physician density was obtained from the Area Health Resources File (AHRF). We conducted multivariate linear regression using 1) average annual melanoma incidence or 2) average annual melanoma mortality by county as primary outcomes, adjusting for demographic confounders and dermatologist density. Cox proportional hazard regression was conducted using individual outcome data from SEER with the same covariates.

**Results:**

Across 611 counties, 167,305 cases of melanoma were analyzed. Per 100,000 people, an additional 10 PCPs per county was associated with 1.62 additional cases of melanoma per year (95% CI 1.06–2.18, p<0.001). This increased incidence occurred disproportionally in early-stage melanoma (Stage 0: 0.69 cases (0.38–1.00), p<0.001; Stage I: 0.63 cases (0.37–0.89), p<0.001; Stage II: 0.11 cases (0.03–0.19), p = 0.005). There was no statistically significant association between PCP density and incidence of stage III or IV melanoma, or with melanoma-specific mortality. Survival analysis demonstrated elimination of 5-year post-diagnosis mortality risk in medically underserved counties after adjusting for stage.

**Conclusions:**

Higher densities of PCPs may be linked to increased diagnosis of early-stage melanoma without corresponding decreases in late-stage diagnoses or melanoma-associated mortality.

## Introduction

Cutaneous malignant melanoma is the deadliest form of skin cancer, a significant contributor to cancer mortality, and carries a substantial and rising public health burden[1]. Relative to other cancers, early detection of melanoma is particularly important to decreasing mortality, as the disease is largely curable at early stages (Stage IA 5-year survival 98%) but resistant to treatment and typically fatal when detected late, with a median survival of unresectable disease of only 5–24 months[2,3]. In addition, the biological course of melanoma initiation and development often provides opportunities for early detection, as most tumors are thought to undergo a prolonged, minimally-invasive radial growth phase prior to progressing to an invasive and potentially morbid vertical growth phase[4].

Traditionally, early detection of cutaneous melanoma has relied on full body skin exams performed by dermatologists; however, skin examination coverage is low in the United States and may be limited by cost, inconvenience, limited availability of specialists outside urban areas, and need for referrals[5]. As such, many patients with suspicious skin lesions will first present to a primary care physician (PCP) during routine clinic visits. PCPs could improve early detection of melanoma in local communities directly, by performing diagnostic biopsies of suspicious skin lesions, or indirectly, by referring patients with need for further evaluation to a dermatologist. However, the population-level health impact of PCP involvement in melanoma detection has been difficult to evaluate[6]. Although PCP visits prior to melanoma diagnosis have been associated with thinner tumors[7], other studies have identified significant obstacles such as a lack of time during clinic visits[8] and the fact that many medical school graduates are not trained to perform skin exams[9]. A pilot primary care screening program in one federal state in Germany initially observed a substantial drop in melanoma mortality in the region of study[10,11], but this success was not replicated when the trial was extended to the national scale[12].

Given these uncertainties, the goal of this study is to better understand the current contribution of PCPs to melanoma detection in the United States by examining the relationship of PCP density with: 1) melanoma incidence by stage, and 2) melanoma-specific mortality, both at the county level. PCP density was chosen due to its association with early detection or improved outcomes in a number of diseases, presumably because the density of PCPs in a given region reflects access to primary care and number of primary care visits in that region[13].

## Methods

All data was obtained in 2016 at the level of the Federal Information Processing Standard (FIPS) county code. Melanoma incidence and mortality data by county was obtained from the Surveillance, Epidemiology, and End Results (SEER) program of the National Cancer Institute (NCI), an authoritative source of population-based information from cancer diagnosis to outcome covering 28% of the US population. County physician supply, demographics, and other characteristics were obtained from the Area Health Resource File (AHRF), which provides a wealth of information about health resources and socioeconomic indicators for all counties in the US. All counties in SEER were included in the analysis, including counties with zero primary melanoma diagnoses, with two exceptions: counties in Alaska (registry only includes Native Alaskan individuals), and Kalawao County in Hawaii (founded for leprosy treatment and administered by Department of Health) were excluded to avoid unintended bias.

The primary outcomes of the study were melanoma incidence (total and subdivided by stage), and melanoma-specific mortality at the county level. These parameters were derived from the SEER dataset and included diagnoses and deaths made in the five-year period from January 2008 to December 2012. Incidence was measured on the tumor-level, including all incident primary melanomas in the study period. Mortality only included deaths attributed specifically to melanoma from 2008 to 2013. Both variables were expressed as rates by county per 100,000 person-years, averaged over the five-year period. Stage was defined by the American Joint Committee on Cancer (AJCC) system, 6^th^ edition.

The primary exposure variable was the number of PCPs in a county per 100,000 population, recorded in the year 2012. We repeated the analysis with 2010 AHRF data, but the findings were unchanged, and the results using the 2012 data are shown throughout. The AHRF data on physician distribution by specialty is derived from the American Medical Association Physician Masterfile.

### Statistical analysis

Effective cancer screening should diagnose cancers at an earlier point in their clinical course, thereby shifting late stage diagnoses to earlier stages. Therefore, we first tested the hypothesis that higher PCP density in a given county would be associated with a higher incidence of earlier stage melanoma and a lower incidence of later stage melanoma.

We estimated separate models for each AJCC clinical stage using county-level melanoma incidence as the dependent variable and PCP density as the independent variable. The following county characteristics were also included in the regression as covariates due to their potential influence: percentage of white non-Hispanic residents, percentage of population over age 65, metropolitan or not (based on U.S. Department of Agriculture urban/rural continuum code[14]), average annual personal income, and density of dermatologists per 100,000 people. An interaction term for PCP density and dermatologist density was also included in the regression to account for the possibility of synergistic effects (through referrals, for example) in detection and diagnosis.

Because a goal of screening and early detection is a reduction in mortality, we also tested the hypothesis that a higher PCP density is associated with a lower melanoma-specific mortality. The SEER disease-specific cause of death classification was used to obtain the melanoma-specific mortality rate in each county over the five-year period from 2008–2012, minimizing potential measurement bias from additional diagnosis codes. For this measure, SEER excludes patients with a prior history of a different cancer type from cause-specific survival analysis. We performed a linear regression using melanoma-specific mortality as the dependent variable and PCP density as the independent variable, including the same covariates.

In order to explore whether post-diagnosis survival on the individual level was inherently different in counties with low PCP density, which could impact interpretation of county-level analyses, we estimated a Cox proportional hazards model using 5-year melanoma-specific survival (i.e. alive or not at 5 years post-diagnosis due to melanoma, as defined previously) as the primary outcome. The same selection criteria from above were applied, but diagnoses from 2003–2012 were included in order to have a sufficient number of patients with 5-years of follow up post-diagnosis. We compared survival of persons residing in Health Professional Shortage Areas (HPSAs)[15] (>3500 residents per 1 PCP, or 28.57 PCPs per 100,000 people) to persons in all other counties included in this study. We also compared survival of individuals residing in one of three county tertiles by PCP density: low (67.50 PCPs per 100,000 people), medium (67.50–91.39), and high (above 91.39), the results of which reflected a similar trend. We used the same covariates, but deriving age and ethnicity from individual records instead of county data.

Because no identifiable data was used in this study, Institutional Board Review was not required as per institutional policy. The analytic code is available from the authors upon request; all analyses were performed using Stata 14 (Statacorp).

## Results

From 2008–2012, a total of 167,305 melanoma diagnoses in 611 counties were reported in SEER. Descriptive county statistics according to participation status in the SEER database are listed in Table 1. The 611 counties included in the study were not notably different in demographic characteristics compared to all 3,142 AHFR counties. Stage information was not reported for 9,329 cases; these were included in analyses of total incidence, but excluded from analyses grouped by stage at diagnosis. Stages 0 and I accounted for 87% of all diagnoses with known stage.

**Table 1:** County-level descriptive statistics, 2008–2012.

Variable	All Included Counties (n = 611)^a^	HPSA Underserved (n = 138)^a^	All AHRF (n = 3,142)^a^
PCP Density^b^	51.6 (30.1, 0–181.73)	14.3 (9.6, 0–28.5)	53.0 (35.5, 0–476)
Dermatologist Density^b^	1.30 (2.44, 0–24.21)	0.09 (0.5, 0–4.9)	1.16 (3.2, 0–109)
Avg Income/Person	36,731 (9923, 17,922–93,407)	32,804 (8109, 19,143–63,434)	37,968 (9809, 17,922–119,347)
Pct Male	49.9 (2.2, 43.0–65.6)	50.3 (2.5, 46.5–65.0)	50.0 (2.2, 43–70)
Pct White non-Hispanic	73.3 (21.1, 10.2–98.4)	76.1 (20.2, 13.1–98.3)	77.7 (19.9, 3.2–98.6)
Pct >65 years old	15.5 (3.9, 3.6–32.0)	16.2 (3.5, 7.5–29.4)	16.7 (4.3, 3.6–49.3)
Pct HS education	82.2 (7.7, 57.4–97.4)	78.7 (8.0, 57.4–94.7)	84.1 (7.0, 44.9–97.5)
Pct Uninsured	21.9 (6.1, 5.7–44.9)	23.7 (5.6, 9.7–44.9)	21.3 (6.6, 3.6–52.3)
Pct of Metropolitan Counties	42.4	37.0	37.1
Melanoma Incidence^b^	23.4 (14.3, 0–115)	19.4 (10.8, 0–58.1)	-
Stage 0	9.48 (7.58, 0–60.2)	7.19 (5.23, 0–25.7)	
Stage I	7.25 (5.71, 0–42.5)	5.97 (5.43, 0–35.4)	
Stage II	2.36 (2.07, 0–19.4)	2.07 (2.14, 0–12.9)	
Stage III	1.22 (1.22, 0–8.95)	1.25 (1.64, 0–8.95)	
Stage IV	0.95 (1.10, 0–8.47)	1.11 (1.60, 0–8.47)	
Melanoma Mortality^b^	2.87 (2.36, 0–26.2)	3.04 (3.30, 0–26.2)	-

^a^ Values displayed as: mean (SD, min-max)

^b^ Per 100,000 people

Abbreviations: primary care provider (PCP), Health Professional Shortage Area (HPSA), Area Health Resource File (AHRF)

### A higher density of PCPs is associated with a higher incidence of melanoma

**Fig 1:**
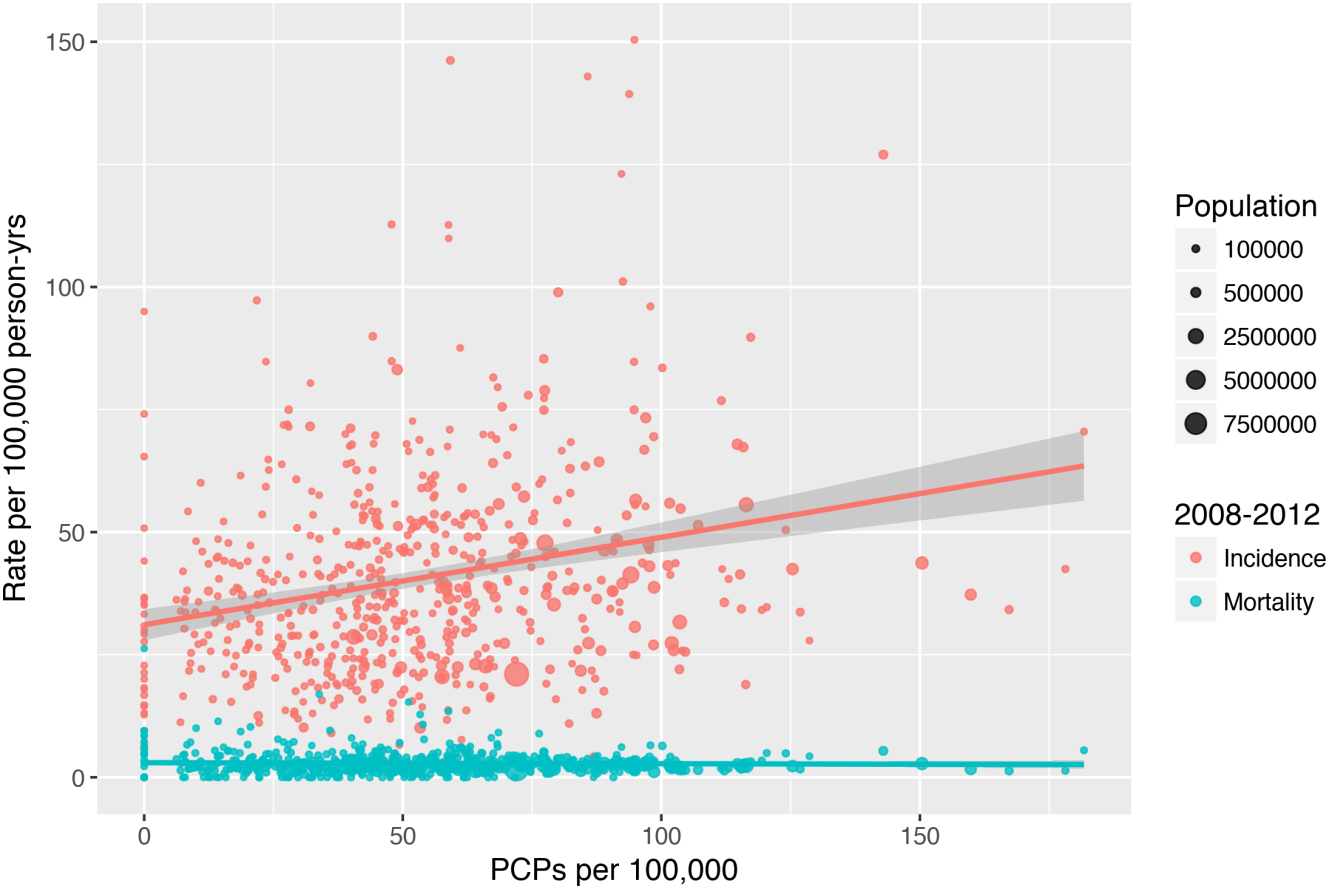
Total incidence and mortality as a function of PCP density. Melanoma incidence per 100,000 person-years and melanoma-specific mortality per 100,000 person-years as a function of PCPs per 100,000 people across all US counties in SEER from 2008–2012. Points are scaled in size by total county population, and the 95% CI for each line of fit is shown in gray.

Fig 1 illustrates a positive relationship between PCP density and overall melanoma incidence. Adjusted models show that higher PCP density of 1 per 100,000 is associated with higher melanoma diagnosis rate of 0.162 per 100,000 (95% CI 0.106–0.218, p<0.001). For example, this model would predict that an increase of 10% in the PCP supply in Los Angeles County would be associated with 116 additional melanoma diagnoses per year, a 5.5% increase over baseline. Among the covariates, higher per capita income, greater percentage of white non-Hispanic residents, greater percentage of elderly residents, status as an urban county, and higher dermatologist density were independently positively associated with incidence (Table 2).

**Table 2:** Multivariate regressions of incidence and mortality with PCP density and co-variates.

	Incidence^a^						Mortality^a^
	Stage 0	Stage I	Stage II	Stage III	Stage IV	All Stages	All Stages
PCP density^b^	0.07 [0.04; 0.10] **p<0.001**	0.06 [0.04; 0.09] **p<0.001**	0.01 [0.00; 0.02] **p = 0.005**	0.00 [-0.00; 0.01] p = 0.32	0.00 [-0.01; 0.00] p = 0.63	0.16 [0.11; 0.22] **p<0.001**	0.00 [-0.01; 0.01] p = 0.91
Income^c^	0.16 [0.07; 0.24] **p<0.001**	0.13 [0.06; 0.21] **p<0.001**	0.00 [-0.02; 0.02] p = 0.98	0.01 [-0.01; 0.02] p = 0.37	0.00 [-0.01; 0.01] p = 0.99	0.29 [0.13; 0.45] **p<0.001**	0.01 [-0.01; 0.03] p = 0.39
Non-Hisp White (%)	0.11 [0.08; 0.15] **p<0.001**	0.16 [0.13; 0.19] **p<0.001**	0.04 [0.03; 0.05] **p<0.001**	0.02 [0.01; 0.02] **p<0.001**	0.01 [0.01; 0.02] **p = <0.001**	0.36 [0.29; 0.42] **p<0.001**	0.14 [0.09; 0.20] **p<0.001**
Age >65 (%)	1.00 [0.78; 1.22] **p<0.001**	0.74 [0.56; 0.93] **p<0.001**	0.14 [0.09; 0.20] **p<0.001**	0.05 [0.01; 0.08] **p = 0.014**	0.03 [0.00; 0.05] p = 0.07	2.15 [1.75; 2.55] **p<0.001**	0.024 [0.02; 0.03] **p<0.001**
Derm density^c^	1.30 [0.53; 2.06] **p<0.001**	0.52 [-0.13; 1.17] p = 0.118	0.02 [-0.18; 0.22] p = 0.85	-0.02 [-0.15; 0.10] p = 0.71	0.00 [-0.10; 0.09] p = 0.95	1.81 [0.41; 3.22] **p = 0.012**	0.04 [-0.15; 0.24] p = 0.68
Urban county	4.39 [2.66; 6.12] **p<0.001**	3.00 [1.53; 4.48] **p<0.001**	0.12 [-0.33; 0.57] p = 0.59	-0.02 [-0.31; 0.27] p = 0.89	0.00 [-0.22; 0.22] p = 0.99	7.40 [4.22; 10.59] **p<0.001**	-0.05 [-0.49; 0.39] p = 0.82
PCP x Derm interaction	-0.01 [-0.02; -0.00] **p = 0.002**	-0.01 [-0.01; 0.00] p = 0.10	0.00 [-0.00; 0.00] p = 0.35	0.00 [0.00; 0.00] p = 0.93	0.00 [0.00; 0.00] p = 0.86	-0.02 [-0.03; 0.00] **p = 0.007**	0.00 [0.00; 0.00] p = 0.67

^a^ Values represent increase in incident cases or deaths per 100,000 persons for incremental increases in respective measures, displayed as coef [95% CI], p-value. For example, for each additional PCP in a county, 0.16 additional melanoma diagnoses are made (both variables per 100,000).

^b^ Physicians per 100,000 persons

^c^ Per capita ($1000)

Boldface indicates statistical significance (p<0.05).

### Higher PCP density is associated with a disproportionate increase in diagnosis of early-stage melanoma and no decrease in melanoma mortality

**Fig 2:**
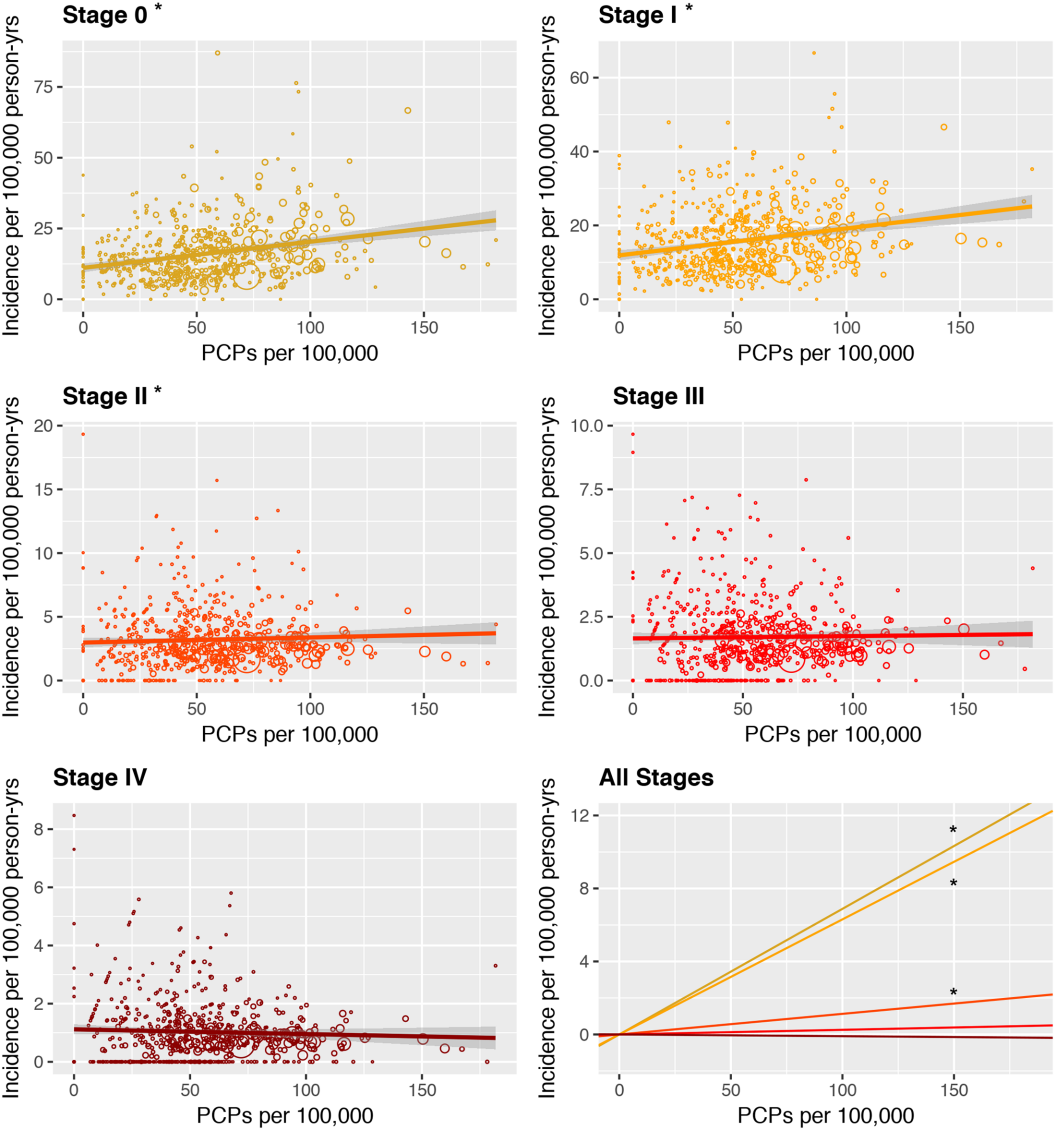
Incidence as a function of PCP density, stratified by stage at diagnosis. Melanoma incidence per 100,000 person-years shown as a function of PCPs per 100,000 people across all US counties in SEER from 2008–2012, split by AJCC stage at diagnosis. Points are scaled in size by total county population, and the 95% CI for each line of fit is shown in gray. Stages 0, I, and II were statistically significant (*), and the last panel compares coefficients for all stages.

Higher PCP density was associated with increasing melanoma diagnosis in stages 0, I, and II; diagnosis rates of stages III and IV melanomas were unrelated to PCP density (Fig 2 and Table 2). This analysis illustrates progressively decreasing coefficients for the association of PCP density with incidence as tumor stage increases, from 0.069 additional stage 0 diagnoses to 0.001 fewer stage IV diagnoses for every additional PCP (Fig 2). Only the regressions for stage 0, I, and II diagnoses were statistically significant at the 0.05 level. PCP density was not associated with melanoma mortality (point estimate 0.0005 fewer deaths per 100,000 person-years for every additional PCP/100,000, 95% CI -0.008–0.007, p = 0.91).

Because traditional mortality analysis counts all deaths due to melanoma in a given year, regardless of the year of the initial diagnosis, it may be inappropriately influenced by remote diagnostic trends. To ensure that we are capturing recent diagnostic patterns, we also calculated an incidence-based mortality rate in which only cases with an initial diagnosis between 2003–2007 were included. Among those cases, deaths from melanoma that occurred from 2003–2012 were counted. This allows for a minimum 5-year follow-up time for all cases while better capturing the incidence patterns during the study period of interest. Using this mortality rate, the association between PCP density and mortality was similarly small and non-significant, when accounting for confounding factors (Coefficient -0.00187, 95% CI -.010–0.006, P = 0.632).

### After adjusting for stage at diagnosis, individuals in more PCP dense counties exhibited no difference in 5-year post diagnosis survival

A Cox proportional hazard regression comparing persons in HPSA counties (>3500 residents per 1 PCP; n = 6,107) to persons in all other included SEER counties (n = 213,938) from 2003–2012, adjusting for sociodemographic confounders but before adjusting for stage, demonstrated a statistically significant decrease in 5-year melanoma-specific survival for persons in HPSAs (Hazard Ratio = 1.18 [1.06; 1.30], p = 0.002). All covariates except dermatologist density also had statistically significant associations with 5-year survival (Table 3). However, after adjusting for AJCC stage at diagnosis, individuals in HPSAs no longer exhibited statistically increased hazard relative to other counties (HR = 0.947 [0.889; 1.01], p = 0.202). Results from a Cox survival analysis using tertiles of PCP density rather than HPSA status exhibited similar trends.

**Table 3:** Cox proportional hazard survival analysis, by HPSA status.

	Original Model		Adjusting for Stage	
	HR [95% CI]	P value	HR [95% CI]	P value
**HPSA Status**				
Not underserved	Reference	--	Reference	--
Underserved	1.18 [1.06; 1.30]	**0.002**	0.947 [0.889; 1.01]	0.202
**AJCC Stage**				
0	--	--	Reference	--
I	--	--	4.30 [3.77; 4.91]	**<0.001**
II	--	--	38.5 [33.9; 43.7]	**<0.001**
III	--	--	102 [90.1; 116]	**<0.001**
IV	--	--	570 [502; 646]	**<0.001**
**Age**	1.03 [1.02; 1.03]	**<0.001**	1.02 [1.02; 1.02]	**<0.001**
**White**	1.76 [1.60; 1.95]	**<0.001**	1.09 [0.976; 1.22]	0.127
**Dermatologist Density**	0.984 [0.962; 1.01]	0.144	0.982 [0.957; 1.00]	0.109
**Metropolitan**	0.929 [0.872; 0.989]	**0.021**	1.05 [0.986; 1.14]	0.115
**Average Income**	0.989 [0.987; 0.991]	**<0.001**	0.995 [0.992; 0.997]	**<0.001**
PCP*Derm Interaction	1.00 [1.00; 1.00]	0.473	1.00 [1.00; 1.00]	0.102

Boldface indicates statistical significance (p<0.05)

## Discussion

Our results suggest that increased PCP density is associated with a higher incidence of earlier stage melanoma, especially for AJCC stages 0 (melanoma in situ), I, and II. These results are consistent with the hypothesis that PCPs play an active role in the diagnosis of melanoma in the United States—perhaps greater than might be expected given the current lack of recommendation from the USPSTF for PCP skin cancer screening[16]. This higher incidence of melanoma in PCP-dense counties is independent of several demographic factors that affect melanoma incidence or vary with PCP density, such as proportion of older residents and ethnic composition. Notably, our regression model directly controlled for density of dermatologists as well as for synergistic effects between PCPs and dermatologists.

Despite the fact that PCP-dense counties show a distinct increase in early-stage melanoma diagnoses, our data show that these counties have no measurable difference in population-level melanoma mortality. To assuage the concern that post-diagnosis survival may be inherently different in PCP dense counties, the survival analysis demonstrates that individual survival is equivalent for persons across counties with the same stage at diagnosis. This combination of evidence is consistent with the phenomenon of overdiagnosis—specifically, the idea that a greater density of PCPs is contributing to diagnoses that do not affect mortality. The clinical diagnosis of indolent malignancies that pose no increased risk of mortality to the patient has been well documented in melanoma as well as other cancer settings, particularly in the context of more intensive efforts to screen for early-stage cancer[17,18].

Several factors are likely contributing to the phenomenon of overdiagnosis in melanoma. Overdiagnosis in melanoma is thought to be directly related to the number of total biopsies performed[19], and PCPs with less training in discriminating melanoma may have a lower threshold for biopsy or for referral for biopsy[20,21]. For pathologists, distinguishing melanoma in situ from benign dysplastic nevi can be challenging[22], and there is data demonstrating a ‘diagnostic drift’ towards a lower threshold for higher grade histopathological diagnoses over time[23,24]. In addition, progression of some lesions may be very slow or even clinically insignificant, with one study reporting that nearly a third of in situ melanomas did not progress to invasive melanoma in a median time of 27 months[25]. Together, these findings suggest that an aggressive treatment approach for clinically ambiguous lesions may not be contributing to improved outcomes, even as utilization rates may be substantial. Given these findings, interventions such as implementation of PCP training programs and systematic methods for determining biopsy and referral thresholds could be studied for the ability to improve late-stage diagnoses and mortality, while also limiting the burden of costs and strain on the healthcare system[26]. Early results in this arena are promising, with pilot screening programs demonstrating increased diagnoses of melanoma without an increase in skin surgeries or referrals[27–29].

This study contributes to an under-developed literature examining the current role that primary care physicians play in the detection of melanoma. Strengths of this study include the population-level design, the high quality validated SEER database, and the ability to examine both incidence by stage and mortality during the same time period. Although the SEER database encompasses only 28% of the national population, analysis of SEER counties reveals that they are highly similar demographically to the country as a whole, relaxing concerns about the representativeness of the SEER population. In addition, the extensive nature of the AHRF database allows for capture of the vast majority of potential confounding county-level variables, including dermatologist density.

A limitation of our study is that it is difficult to detect modest impacts on stage IV diagnoses or mortality due to the relative rarity of these events. However, our point estimates for incidence and mortality were centered at 0, and the narrow width of our standard errors suggests that we would be powered to detect larger effects, mitigating concern for a possible Type II error. Additional limitations include the necessary use of PCP density as a surrogate for number of primary care visits, as well as the possibility of a ‘spillover’ effect for adjacent counties due to patients traveling outside of their county of residence for medical care, especially if there are few physicians. The ecologic and cross-sectional study design inherently limits inferences about causality. Furthermore, as with any retrospective analysis using data that were not collected to answer this specific research question, there is always the possibility of unobserved confounding variables, missing data, and misclassification bias.

## Conclusions

We find evidence that the availability of PCPs is associated with increased early-stage melanoma diagnosis but not mortality, independent of the availability of dermatologists, and accounting for county-level factors associated with melanoma risk and diagnosis. Though causality was not formally assessed, this relationship raises consequential clinical questions and suggests a need to re-examine the use of primary care resources with regard to skin cancer detection. Additional research regarding rates and outcomes of diagnostic skin procedures in primary care settings is warranted.
